# Effects of Icariin on Reproductive Functions in Male Rats

**DOI:** 10.3390/molecules19079502

**Published:** 2014-07-03

**Authors:** Maoxin Chen, Jie Hao, Qiaozhen Yang, Gang Li

**Affiliations:** 1 Institute of Life Sciences, Chongqing Medical University, Chongqing 400016, China; E-Mails: chenmaoxin1989@163.com (M.C.); 31358123@163.com (Q.Y.); 2 The First Affiliated Hospital, Chongqing Medical University, Chongqing 400016, China; E-Mail: haoj_cq@sina.com

**Keywords:** icariin, male reproductive functions, testosterone, Sertoli cells, Leydig cells

## Abstract

The present study investigated the effects and potential mechanism(s) of action of icariin on the reproductive functions of male rats. Adult rats were treated orally with icariin at doses of 0 (control), 50, 100, or 200 mg/kg body weight for 35 consecutive days. The results show that icariin had virtually no effect on the body weight or organ coefficients of the testes or epididymides. However, 100 mg/kg icariin significantly increased epididymal sperm counts. In addition, 50 and 100 mg/kg icariin significantly increased testosterone levels. Real-time PCR suggests icariin may be involved in testosterone production via mRNA expression regulation of genes such as peripheral type benzodiazepine receptor (PBR) and steroidogenic acute regulatory protein (StAR). Furthermore, 100 mg/kg icariin treatment also affected follicle stimulating hormone receptor (FSHR) and claudin-11 mRNA expression in Sertoli cells. Superoxide dismutase (SOD) activity and malondialdehyde (MDA) levels were measured in the testes; 50 and 100 mg/kg icariin treatment improved antioxidative capacity, while 200 mg/kg icariin treatment upregulated oxidative stress. These results collectively suggest that icariin within a certain dose range is beneficial to male reproductive functions; meanwhile, higher doses of icariin may damage reproductive functions by increasing oxidative stress in the testes.

## 1. Introduction

Icariin (C_33_H_40_O_15_; molecular weight: 676.67), a flavonoid isolated from *Herba epimedii*, is considered to be the main active component responsible for the actions of the plant*.* *H. epimedii* has been proven to be an effective remedy for cardiovascular diseases, osteoporosis, and tumors; moreover, and it can improve endocrine, immune, and cognitive functions [1,2,3,4,5,6,7,8]. In addition, *H. epimedii* has been traditionally used in China to treat erectile dysfunction [9]. Animal experiments show that icariin administration improves erectile function in aged male rats [10] as well as streptozotocin-induced diabetic rats [11]. Phosphodiesterase-5 (PDE5) inhibitors including sildenafil, vardenafil, and tadalafil are synthetic compounds that are currently first-line treatments for erectile dysfunction. PDE5 inhibitors bind to the cGMP-catalytic site on PDE5, preventing it from destroying cGMP. The resultant accumulation of cGMP in penile smooth muscle cells allows patients with erectile dysfunction to have an otherwise unattainable erection. Furthermore, the clinical effect of a PDE5 inhibitor requires at least a minimal nitric oxide signal triggered by sexual stimulation. Icariin significantly inhibits PDE-5 and induces nitric oxide synthase expression in corpus cavernosum smooth muscle [12,13,14,15]; this could at least partly account for the effect of icariin on erectile function. 

Previous studies on the pharmacological effects of icariin on reproductive functions mainly focus on its putative role in potentiating sexual function. Meanwhile, few studies have investigated its other pharmacological activities on reproductive functions such spermatogenesis and testosterone production or its underlying mechanisms. Furthermore, its potential adverse effects on the testes remain to be investigated. Therefore, in the present study, we treated adult rats with different doses of icariin and analyzed the effects on male reproductive functions as well as underlying mechanisms by using histological examinations, sperm count, ELISA, and real-time PCR. 

## 2. Results and Discussion

### 2.1. Effects of Icariin on Body Weight

**Figure 1:**
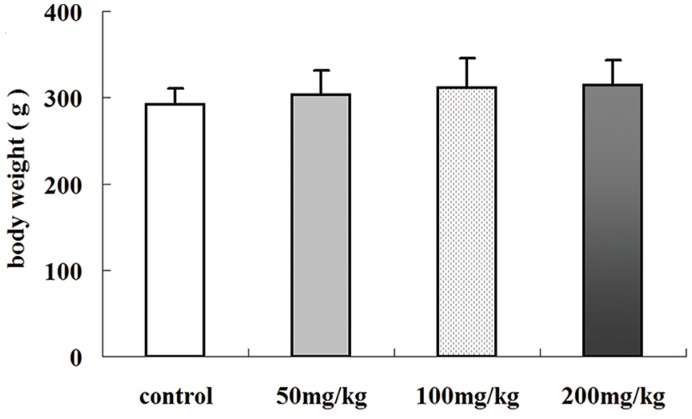
Effects of icariin on the body weight of SD rats after treated with different doses of icariin. Values are the mean ± SD of 10 rats.

Male Sprague–Dawley (SD) rats were exposed to different doses of icariin, and body weight was measured weekly. As shown in Figure 1, body weight did not change significantly in any group (*p* > 0.05), suggesting icariin has no obvious effects on adult rat growth, which is concordant with previous reports [16,17].

### 2.2. Effects of Icariin on Organ Coefficients

**Figure 2:**
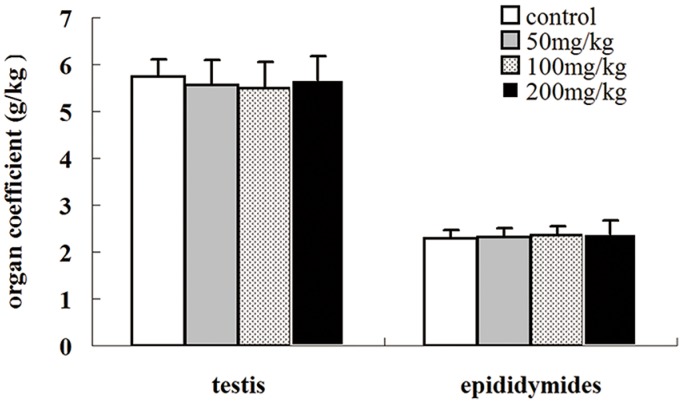
Effects of icariin on the coefficients of the testes and epididymides of SD rats treated with different doses of icariin. Values are the mean ± SD of 10 rats.

The effects of icariin on the coefficients of the testes and epididymides were also examined. As shown in Figure 2, icariin treatment did not significantly alter the organ coefficients of the testes or epididymides compared to the control (*p* > 0.05). 

### 2.3. Effects of Icariin on Testicular Morphology

**Figure 3:**
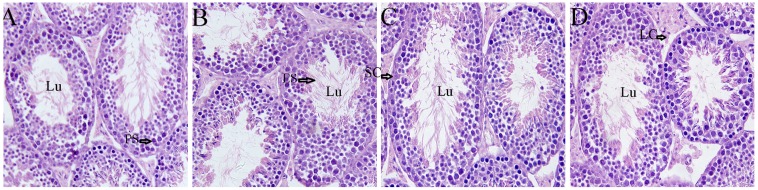
Histology of the testes in SD rats after treatment with different doses of icariin (×200). (**A**) Control, (**B**) 50 mg/kg icariin, (**C**) 100 mg/kg icariin, (**D**) 200 mg/kg icariin. No histopathological alterations in the testes were observed in the control or icariin-treated groups. Lu, lumen; Sc, Sertoli cells; Lc, Leydig cells; PS, primary spermatocyte; ES, early spermatid.

No histopathological alterations in the testes were observed in the control or icariin-treated groups. Leydig and Sertoli cells appeared normal (Figure 3). The base membrane of the seminiferous tubules was continuous and complete, and germ cells had normal morphology and regular arrangement. These findings suggest the 3 doses of icariin did not adversely affect testis histopathology.

### 2.4. Icariin-Induced Changes in Epididymal Sperm Count

**Figure 4:**
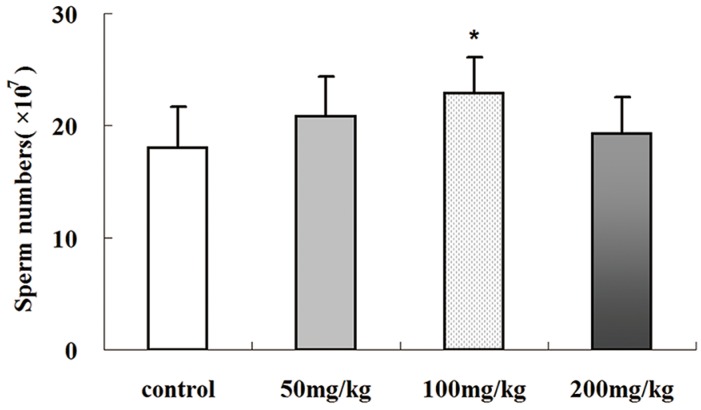
Epididymal sperm counts in SD rats treated with different doses of icariin. Values are the mean ± SD of 10 rats. * *p<* 0.01 *vs.* control group.

As shown in Figure 4, 100 mg/kg icariin treatment resulted in higher sperm count than the control group (*p* < 0.01). Both the 50 and 200 mg/kg icariin groups had slightly higher sperm counts than the control group, but neither showed a significant difference (*p* > 0.05). This indicates icariin has dose-related effects on spermatogenesis. 

### 2.5. Effects of Icariin on Serum Testosterone Production

**Figure 5:**
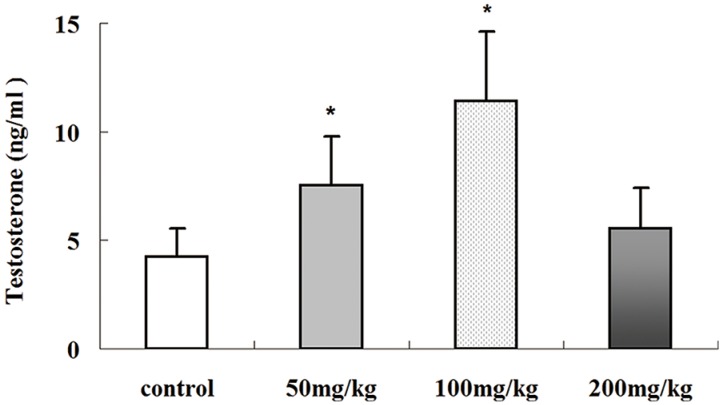
Effects of icariin on serum testosterone levels in male SD rats. Serum testosterone concentrations were evaluated individually in all groups of rats by ELISA. Values are the mean ± SD of 10 rats. * *p <* 0.01 *vs.* control group.

One main function of Leydig cells in the testes is testosterone production. Icariin has testosterone mimetic properties [18] and may increase testosterone secretion in Leydig cells by affecting cAMP *in vitro* [19]. In the present study, the serum testosterone concentrations of icariin treated-rats were detected by ELISA (Figure 5). Testosterone levels increased significantly with increasing icariin dose from 50 to 100 mg/kg (*p* < 0.01). However, no statistically significant difference was detected between the control and 200 mg/kg icariin-treated group (*p* > 0.05), suggesting higher doses of icariin do not increase testosterone production.

### 2.6. Effects of Icariin on mRNA Expression Levels of Luteinizing Hormone Receptor (LHR) and Steroidogenic Genes

**Figure 6:**
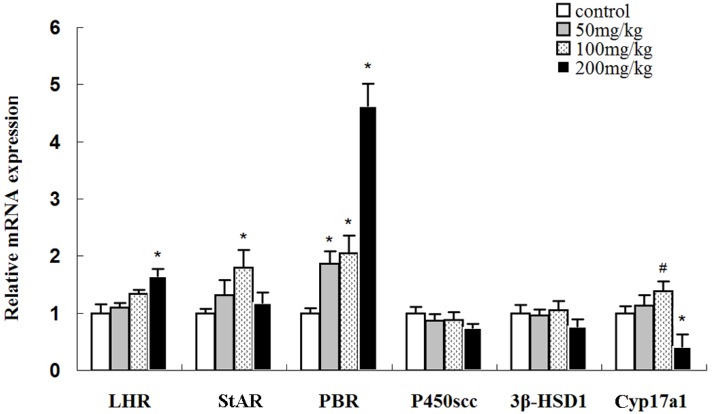
Expressions of LHR, P450SCC, Cyp17a1, PBR, StAR, and 3β-HSD1 mRNA in the testes of rats treated with different doses of icariin. The mRNA levels were normalized to β-actin mRNA. The mRNA levels of the control group were set as 1. Values are the mean ± SD of 10 rats. ^#^ *p <* 0.05, * *p <* 0.01 *vs.* control group.

To preliminarily investigate the possible mechanisms underlying the effect of icariin on testosterone production, the mRNA levels of LHR and several specific genes encoding enzymes involved in the conversion of cholesterol to testosterone were analyzed by real-time PCR (Figure 6). Luteinizing hormone (LH), which is secreted by the pituitary gland, can increase testosterone production by affecting Leydig cells in the testes. The binding of LH with LHR activates adenylate cyclase, subsequently increasing cAMP levels in Leydig cells, which triggers a cascade of intracellular events and eventually testosterone production in Leydig cells [20,21]. In the present study, the 50, 100, and 200 mg/kg icariin-treated groups tended to have higher LHR levels than the control group, but the increase was only significant the 200 mg/kg icariin-treated group. However, 200 mg/kg icariin treatment did not increase testosterone production. Thus, it seems icariin does not affect testosterone production through the regulation of LHR mRNA expression. 

Transmembrane transport of cholesterol from the mitochondrial outer membrane to the inner membrane is the key step in testosterone production that is regulated by cholesterol transmembrane transport regulators including PBR [22] and StAR [23]. Real-time PCR showed that icariin upregulated PBR mRNA in a dose-dependent manner. Meanwhile, StAR mRNA expression exhibited a different pattern: StAR was significantly upregulated in the 100 mg/kg icariin-treated group (*p* < 0.05) but normal in the 200 mg/kg icariin-treated group (*p* > 0.05). The results suggest 50 and 100 mg/kg icariin may increase testosterone production by upregulating StAR and PBR mRNA expression, which in turn result in increased cholesterol transmembrane transport. Both StAR and PBR are indispensable elements of the steroidogenic machinery and function in a coordinated manner to transfer cholesterol into mitochondria; the finding that 200 mg/kg icariin treatment did not affect testosterone levels could at least partly be due to StAR gene expression returning to normal.

Cytochrome P450 side chain cleavage (P450scc) is the only enzyme that converts cholesterol into pregnenolone. The results show that icariin had no or very little effect on P450scc mRNA expression (*p* > 0.05). 3β-hydroxysteroid dehydrogenase type 1 (3β-HSD1) and cytochrome P450 17a1 (Cyp17a1) are also involved in testosterone production [23,24]. 3β-HSD1 mRNA did not change significantly in any icariin-treated group, while Cyp17a1 mRNA expression level was significantly upregulated in the 100 mg/kg icariin-treated group (*p* < 0.05) and significantly downregulated in the 200 mg/kg icariin-treated group (*p* < 0.01).

A comprehensive analysis of all the above-mentioned steroidogenic gene expression levels suggests that an appropriate dose of icariin can increase testosterone production via the icariin-mediated upregulation of StAR, Cyp17a1, and PBR expression. However, when the dose of icariin was further increased to 200 mg/kg, testosterone production did not increase, possibly due to StAR gene expression returning to normal as well as the significant downregulation of Cyp17a1, although LHR and PBR were further upregulated. 

### 2.7. Effects of Icariin on the mRNA Expression Levels of Several Sertoli Cell-Specific Genes

**Figure 7:**
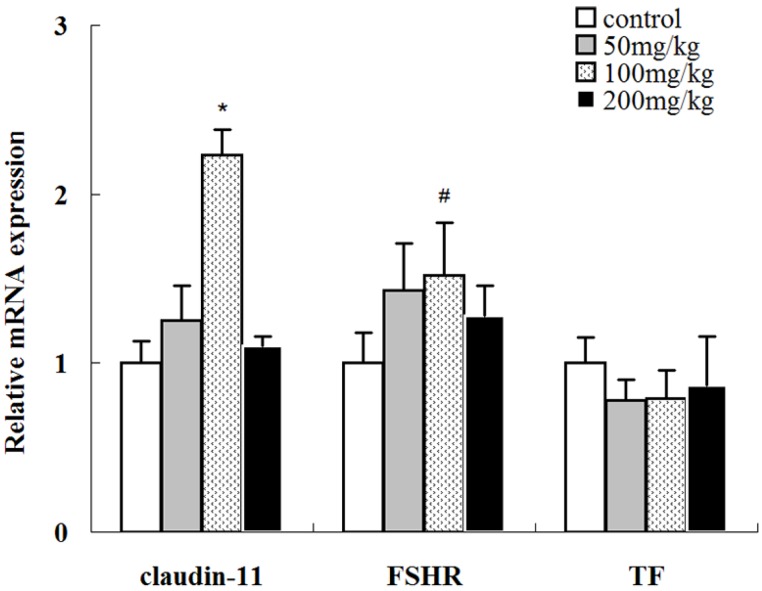
Expressions of claudin-11, FSHR, and TF mRNA in the testes of rats treated with different doses of icariin. The mRNA levels were normalized to β-actin mRNA. The mRNA levels of the control group were set as 1. Values are the mean ± SD of 10 rats. ^#^ *p <* 0.05, * *p <* 0.01 *vs.* control group.

The expression levels of several well-known spermatogenesis-related genes including transferrin (TF), FSHR, and claudin-11, which are expressed in the Sertoli cells of the testes, were evaluated to further understand the effects of icariin on the reproductive functions of rats (Figure 7). 

TF produced by Sertoli cells transports iron to spermatogenic cells so they can meet their developmental requirement [25,26]. TF mRNA level remained unchanged following icariin treatment (*p* > 0.05).

FSHR mRNA expression was upregulated with 50 and 100 mg/kg icariin treatment in a dose-dependent manner (*p* < 0.05) while it was close to normal upon 200 mg/kg icariin treatment. Postnatal testicular development depends on gonadotropin and androgen stimulation. Follicle-stimulating hormone (FSH) acts through FSHR on Sertoli cells to stimulate spermatogenesis [27,28]. FSH regulates the proliferation and activity of Sertoli cells during postnatal development [29]. FSHR ablation reduces the number of Sertoli and germ cells, indicating it can affect spermatogenesis [30]. In the present study, 100 mg/kg icariin treatment increased sperm count, which could be partly due to the increase in the FSHR mRNA level. 

In adult mammals such as rats, the blood–testis barrier (BTB) is mainly formed by the tight junctions among Sertoli cells and adherens junctions between Sertoli cells and germ cells. The BTB divides the seminiferous epithelium into the basal and abluminal compartment [31]. Recent studies show that cytokines such as TGF-β3 and TNFα regulate the steady-state levels of integral membrane proteins (e.g., occludin, ZO-1, and claudin-11) at the BTB, thereby affecting BTB integrity and further affecting spermatogenesis [32,33]. In the present study, claudin-11 expression was significantly upregulated in the 100 mg/kg icariin-treated group (*p* < 0.01) but not in the other groups. These results suggest 100 mg/kg icariin may affect spermatogenesis by affecting the tight junction permeability barrier between Sertoli cells, possibly mediated via its effects on claudin-11 mRNA expression.

### 2.8. Effects of Icariin on SOD Activities and MDA Levels in Rat Testis

The antioxidation defense system can inhibit lipid peroxidation or degrade peroxides to remove excessive oxygen free radicals, protecting the organism from oxidative damage. SOD activity and MDA level roughly reflect tissue oxidative balance [2,34]. Icariin has an antioxidative effect [35,36], protecting tissues and organs through resistance to high oxidative damage. 

Therefore, we determined if icariin regulates the oxidative balance in the testes by analyzing SOD activity and MDA levels. SOD activity increased gradually in the testicular tissues in the 50 and 100 mg/kg icariin groups (*p <* 0.01) (Table 1). However, when the dose was further increased to 200 mg/kg, SOD activity was not significantly different from that in the control group. MDA levels were significantly downregulated and upregulated in the 50 and 200 mg/kg groups, respectively (*p <* 0.01). The results indicate that 50 and 100 mg/kg icariin treatment can improve the testes’ antioxidative ability and reduce lipid peroxidation ability. Meanwhile, 200 mg/kg icariin treatment augments oxidative stress, indicating icariin may exert anti- or pro-oxidative effect in the testes depending on the dose administered. 

**Table 1:** Effects of icariin on SOD activity and MDA level.

Groups	SOD (U/mg)	MDA (nmol/ mg)
Control	96.1 ± 10.84	3.47 ± 0.3
50 mg/kg 100 mg/kg	115.83 ± 17.45 * 123.53 ± 25.65 *	3.1 ± 0.32 ^#^ 3.55 ± 0.33
200 mg/kg	103.73 ± 20.21	4.12 ± 0.47 *

Values are the mean ± SD of 10 rats. ^#^ *p <* 0.05, * *p <* 0.01 *vs.* control group.

The effects of 200 mg/kg icariin on sperm count, testosterone production, and the oxidative balance in the testes collectively indicate that an excessive dose of icariin does not improve male reproductive functions; on the contrary, it may cause adverse effects such as tissue and organ oxidative damage as well as further damage to reproductive functions. Thus, the use of high doses of icariin in disease treatment should take into account its potential adverse effects on reproductive functions.

## 3. Experimental

### 3.1. Materials

**Figure 8:**
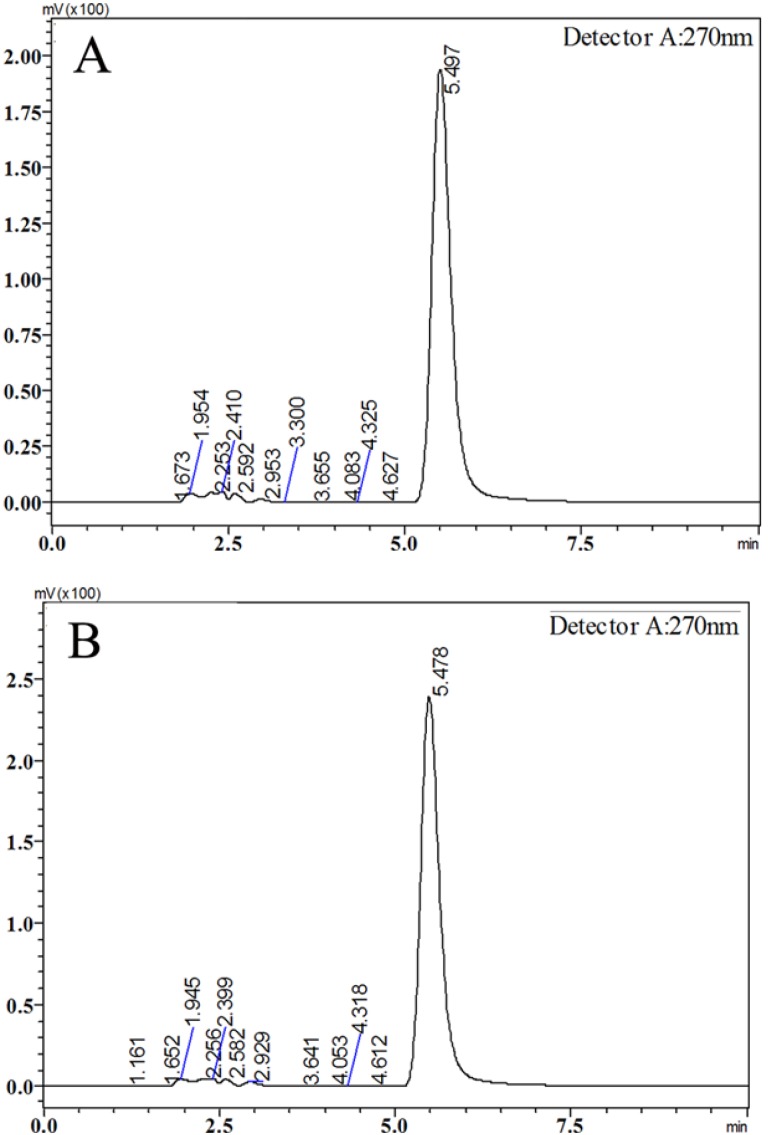
Representative chromatograms of icariin standard (**A**) and icariin sample (**B**).

Icariin (purity > 98%) was purchased from the Nanjing TCM Institute of Chinese Materia Medica (Nanjing, China). The chromatographic peak of icariin was confirmed by comparing its retention time and UV spectra with corresponding icariin standard (Figure 8). The purity of icariin was assessed by the area normalization method. The SYBR^®^ PrimeScript^®^ RT-PCR Kit (Perfect Real Time) was purchased from TaKaRa Biotech (Liaoning, China). Commercial kits used for the analyses of SOD and MDA were purchased from the Beyotime Institute of Biotechnology (Jiangsu, China). Testosterone immunoassay kits were obtained from USCNK (Wuhan, China). All other chemicals were purchased from Sangon Biotech (Shanghai, China). 

### 3.2. Animal Experiments

Forty adult male SD rats weighing 200–290 g (12–16 weeks old) were purchased from the Experimental Animal Centre of Chongqing Medical University. The rats were randomly assigned to groups (*n =* 10 per group) according to their body weight. Icariin was dissolved in DMSO and diluted in PBS to make working solution. The rats received daily intragastric administration of icariin at 0 (control), 50, 100, or 200 mg kg^−1^ day^−1^ for 35 consecutive days. The animals were weighed weekly, and the treatments were adjusted accordingly. At the end of the icariin treatment period, all rats were sacrificed; blood samples were subsequently collected for further analyses of testosterone levels. The animals were handled according to a protocol approved by the Animal Care and Use Committee of Chongqing Medical University.

### 3.3. Relative Weights of Reproductive Organs

After the rats were sacrificed, the reproductive organs including the testes and epididymides were weighed on an electronic balance. The organ coefficients of these organs were subsequently determined as follows: organ coefficient = organ weight/body weight × 1,000.

### 3.4. Epididymal Sperm Count

The right epididymis was used for sperm count. Epididymal sperm were collected by cutting the epididymides into small pieces, which were then immersed in PBS, and subsequently counted using a hemocytometer (×10^7^/mL). 

### 3.5. Histopathological Examination

The testes were removed and fixed in 10% formaldehyde solution at room temperature. The samples were dehydrated with an ethanol gradient, treated with dimethylbenzene, embedded in paraffin, cut into 5-μm-thick sections, and stained with hematoxylin-eosin. Changes in testicular morphology and structure were observed by microscopy. 

### 3.6. Detection of Serum Testosterone

Serum testosterone levels were detected by using an ELISA kit according to the manufacturer’s instructions.

### 3.7. Real-Time PCR

The expression levels of LHR, P450SCC, Cyp17a1, PBR, StAR, 3β-HSD1, claudin-11, FSHR, and TF mRNA in the testicular tissues were assessed by real-time RT-PCR. The primers are listed in Table 2.

**Table 2:** Primer sequences used in RT-PCR analysis.

Gene	Sequence	Length	References
Actin	ACGTTGACATCCGTAAAGAC	200 bp	
	GAAGGTGGACAGTGAGGC		
PBR	ACACTGGTCAGCTGGCTCTGAA	175 bp	[37]
	CAGGCCAGGTAAGGATACAGCAA		
StAR	GGGCATACTCAACAACCAG	111 bp	[38]
	ACCTCCAGTCGGAACACC		
LHR	CATTCAATGGGACGACTCTA	130 bp	[38]
	GCCTGCAATTTGGTGGA		
P450SCC	AGTATCCGTGATGTGGGG	125 bp	[38]
	CATACAGTGTCGCCTTTTCT		
Cyp17a1	GCAGAGTTACTTGCCCTTCGG	142 bp	[38]
	CAGGCGGGGCAGTTGTTTAT		
3β-HSD1	TGTGCCAGCCTTCATCTAC	145 bp	[38]
	CTTCTCGGCCATCCTTTT		
Claudin-11	GCTTCGTGGGTTGGAT	82 bp	
	CAGGTGGGGATGGTGTA		
TF	GCTGTGGCCAGTTTCTTCTC	163 bp	[39]
	CCACATCTCCACCTCCATCT		
FSHR	GGCCAGGTCAACATACCGCTTG	162 bp	

Briefly, total RNA from the rat testes was extracted using TriZol reagent according to the manufacturer’s instructions. First-strand cDNA was synthesized using a SYBR^®^ PrimeScript^®^ RT-PCR Kit (Perfect Real Time) in a thermal cycler (CFX96, Bio-Rad, Hercules, CA, USA). Each real-time PCR reaction was carried out in triplicate in a 20-μL reaction mixture (2 μL cDNA, 6.8 μL H_2_O, 10 μL SYBR^®^ Premix Ex Taq™, and 0.6 μL of each 10 μM forward and reverse primers). The PCR program was 1 min at 95 °C followed by 30 cycles of 10 s at 95 °C, 30 s at 60 °C, and 30 s at 72 °C. The data were analyzed according to the 2^−ΔΔCt^ method. The mRNA expression levels were normalized to a concurrent measurement of β-actin mRNA levels.

### 3.8. Determination of SOD Activity and MDA Level

The testicular tissues were homogenized in 10× ice-cold PBS (w/v) and centrifuged at 4000 rpm for 15 min. The supernatant was used to determine for SOD activity and MDA level. The experimental procedures for determining the SOD activity and MDA level were based on the protocols provided by the manufacturer. SOD activity was determined by the xanthine/xanthine oxidase method. One unit of SOD activity was defined as the amount of protein that inhibited the rate of NBT reduction by 50%. MDA levels were measured by analyzing the reaction of MDA with thiobarbituric acid (TBA); this forms a MDA-TBA2 adduct that absorbs strongly at 535 nm. MDA levels are expressed as per milligram protein.

### 3.9. Statistical Analysis

The data are expressed as mean ± SD The significance of differences was evaluated by using one-way ANOVA. The level of significant was set at *p* < 0.05.

## 4. Conclusions

Icariin has a wide range of effects on reproductive functions in male rats. We, for the first time, report that an appropriate dose of icariin can increase testosterone production by regulating the expressions of genes such as StAR and PBR; icariin can also affect spermatogenesis by regulating FSHR and claudin-11 mRNA expression. However, an excessive dose of icariin may cause adverse effects such as tissue and organ oxidative damage, consequently damaging reproductive functions.
